# Summer is not associated with higher live birth rates in fresh IVF/ICSI cycles: a population-based nationwide registry study

**DOI:** 10.1093/hropen/hoac036

**Published:** 2022-08-24

**Authors:** Eva Carlsson Humla, Christina Bergh, Randa Akouri, Panagiotis Tsiartas

**Affiliations:** Department of Obstetrics and Gynecology, Reproductive Medicine, Sahlgrenska University Hospital, Gothenburg, Sweden; Department of Obstetrics and Gynecology, Reproductive Medicine, Sahlgrenska University Hospital, Gothenburg, Sweden; Department of Obstetrics and Gynecology, Institute of Clinical Sciences, Sahlgrenska Academy, University of Gothenburg, Gothenburg, Sweden; Department of Obstetrics and Gynecology, Reproductive Medicine, Sahlgrenska University Hospital, Gothenburg, Sweden; Department of Obstetrics and Gynecology, Institute of Clinical Sciences, Sahlgrenska Academy, University of Gothenburg, Gothenburg, Sweden; Department of Obstetrics and Gynecology, Institute of Clinical Sciences, Sahlgrenska Academy, University of Gothenburg, Gothenburg, Sweden; Nordic IVF & Gynecology Stockholm, Solna, Sweden

**Keywords:** IVF/ICSI, seasons, live birth rate, clinical pregnancy rate, miscarriage rate, pregnancy outcome

## Abstract

**STUDY QUESTION:**

Is summer associated with a higher live birth rate after fresh IVF/ICSI?

**SUMMARY ANSWER:**

There was no support for a higher live birth rate after fresh IVF/ICSI when treatment was performed during the summer season.

**WHAT IS KNOWN ALREADY:**

Seasonal variations in human natural conception and birth rates are well described. It has been hypothesized that serum vitamin D, levels of which are associated with sun exposure, may have a role in human natural conception rates. However, the association between seasons and IVF outcomes has not yet been clarified and conflicting reports have been published. Furthermore, it has been suggested that women with normal vitamin D levels have a better pregnancy outcome after ART compared to those with vitamin D insufficiency.

**STUDY DESIGN, SIZE, DURATION:**

A nationwide, register-based cohort study including all first-time fresh IVF/ICSI treatments (n = 52 788) leading to oocyte retrieval in Sweden between 2009 and 2018 was carried out.

**PARTICIPANTS/MATERIALS, SETTING, METHODS:**

All first-time fresh IVF/ICSI cycles leading to oocyte retrieval were identified in the National Quality Registry of Assisted Reproduction. Data collected included patient characteristics as well as information about the treatment cycle and pregnancy outcome. The patients were divided into season subgroups, (summer, autumn, winter and spring) based on the date of oocyte retrieval. The primary outcome was live birth rate, which was defined as the number of live births per oocyte retrieval and embryo transfer (ET). Other outcomes included clinical pregnancy per ET and miscarriage per clinical pregnancy. Logistic regression with multiple imputation was performed to evaluate whether there was an association between season and IVF/ICSI outcomes, with summer as reference. Adjustments were made for woman’s age, year of treatment, BMI, total FSH/hMG dose, type of treatment, fertilization type, embryonic stage at ET and number of embryos transferred.

**MAIN RESULTS AND THE ROLE OF CHANCE:**

Live birth rate per oocyte retrieval ranged between 24% and 26% among seasons. A significantly higher live birth rate was seen for spring compared with summer, 26% versus 24%, respectively (adjusted odds ratio (OR) 1.08, 95% CI 1.02–1.16, *P *=* *0.02). No significant association was seen when winter and autumn were compared with summer. Live birth rate per ET ranged between 29% and 31% among seasons. A significantly higher live birth rate was seen for spring and autumn compared with summer, at 31% and 31%, respectively versus 29% (adjusted OR 1.08, 95% CI 1.01–1.16, *P *=* *0.04 and adjusted OR 1.09, 95% CI 1.01–1.16, *P *=* *0.02), respectively. No significant association was seen when winter was compared with summer. Clinical pregnancy rate varied between 36% and 38% and miscarriage rate between 16% and 18%, with no significant seasonal associations.

**LIMITATIONS, REASONS FOR CAUTION:**

Possible limitations are the retrospective design of the study and unmeasured confounders. Another limitation is that a generalized estimating equation (GEE) model was not used. The use of a GEE model would have made it possible to include all started fresh IVF/ICSI cycles since it allows for correction for any dependence between cycles within women.

**WIDER IMPLICATIONS OF THE FINDINGS:**

The results of this large registry study give no support for the hypothesis that IVF/ICSI treatments performed during summer season, with the highest degree of sunlight and vitamin D synthesis, is associated with higher pregnancy and live birth rates. In fact, our results showed significantly lower live birth rates during summer compared with spring and autumn. However, the magnitude of this difference was small and unlikely of clinical value. We suggest that season should not be taken into consideration when planning and performing fresh IVF/ICSI treatments.

**STUDY FUNDING/COMPETING INTEREST(S):**

Financial support was received through the Swedish state under the agreement between the Swedish government and the county councils, the ALF-agreement (ALFGBG-70 940) and grants from the Hjalmar Svensson’s Research Foundation (HJSV2021019 and HJSV2021037). None of the authors declare any conflict of interest.

**TRIAL REGISTRATION NUMBER:**

N/A.

WHAT DOES THIS MEAN FOR PATIENTS? This study looked at whether summer is associated with a higher live birth rate after IVF/ICSI. It is known that the number of births after natural conception shows seasonal variations associated with environmental temperature and light exposure, but the association between seasons and IVF/ICSI outcomes has not yet been clarified. Levels of vitamin D in blood vary with sun exposure, and this may play a role in human natural conception rates; however, it is unclear whether this is also the case in assisted reproduction. This large study included more than 52 000 women with infertility who underwent their first IVF/ICSI treatment with fresh embryo transfer (ET) in Sweden from 2009 to 2018. The women were identified from the national registry. The women were divided into seasonal subgroups: summer, spring, autumn and winter. We found that live birth rate per oocyte retrieval varied between 24% and 26% and was higher in spring than summer. Live birth rate per ET was 29–31%, and spring and autumn had higher rates than summer. There were no differences between seasons when looking at the rate of pregnancies confirmed by ultrasound 7–9 weeks after ET (presence of a gestational sac) or miscarriage rate. Our study showed that slightly higher live birth rates occur when IVF/ICSI takes place in spring and autumn compared to summer. However, the clinical impact of this difference is probably negligible and we suggest that season should not be taken into consideration when planning and performing IVF/ICSI.

## Introduction

Seasonal variations in human natural conception and birth rates have been demonstrated by several studies (James, 1990; Rojansky *et al.*, 1992; Lam and Miron, 1994; Cummings, 2014; Wesselink *et al.*, 2020). It has been hypothesized that natural conception is affected by biological factors, such as environmental temperature and light exposure, which possibly affect sperm quality, melatonin secretion and vitamin D synthesis, all important parameters for the reproductive efficacy (Kauppila *et al.*, 1987; Lam and Miron, 1991; Rojansky *et al.*, 1992; Reiter, 1993; Levine, 1994; Bronson, 1995; Levine, 1999; Cummings, 2003, 2007, 2010a,b; Reiter *et al.*, 2014; Blomberg Jensen *et al.*, 2018). However, a decline in seasonal variation in births has occurred in the last few decades in several European countries, including Sweden (Lerchl *et al.*, 1993; Russell *et al.*, 1993; Cancho-Candela *et al.*, 2007; Haandrikman and van Wissen, 2008; Régnier-Loilier, 2010; Björnsson and Zoega, 2017; Dahlberg and Andersson, 2018, 2019; Cypryjański, 2019). Although the reasons for these changes remain difficult to determine, cultural behaviours and sociodemographic decisions seem to play an important role in the seasonal timing of childbearing (Odegård, 1977; James, 1990; Bobak and Gjonca, 2001; Dahlberg and Andersson, 2018).

The association between seasonal variation and IVF outcomes has not yet been clarified, and conflicting reports have been published. It has been hypothesized that the increased synthesis and higher blood levels of vitamin D, which are associated with sun exposure during summer months, may influence ART outcomes (Cozzolino *et al.*, 2020; Iliuta *et al*., 2022). Since most variables influencing human reproductive activity and success are controlled during IVF, the use of ART as a model for studying the relationship between seasonality and reproductive outcomes could be considered optimal.

Several small, retrospective studies from Brazil, Israel, Belgium and England that included between 1074 and 9865 IVF cycles have suggested that there is seasonal variation with better outcomes in terms of number of retrieved mature oocytes, fertilization rate, embryo quality, implantation rate and clinical pregnancy rate, when IVF treatment was performed during spring and summer (Rojansky *et al.*, 2000; Wood *et al.*, 2006; Braga *et al.*, 2012; Vandekerckhove *et al.*, 2016). One study from Belgium that included 9865 fresh IVF cycles demonstrated higher live birth rates after IVF treatments were performed during summer (Vandekerckhove *et al.*, 2016). However, other studies from Israel, Italy and Switzerland including between 2067 and 7368 IVF cycles could not confirm the presence of any seasonal variations in implantation rate and clinical pregnancy rates after IVF (Fleming *et al.*, 1994; Gindes *et al.*, 2003; Revelli *et al.*, 2005; Wunder *et al.*, 2005; Kirshenbaum *et al.*, 2018). One recent cohort study from China including 13 223 fresh IVF cycles showed no association between seasons and live birth rate after fresh embryo transfer (ET) (Liu *et al.*, 2019).

Since there are conflicting data and a lack of studies from high latitude countries, where day length is highly variable during the year, we aimed to elucidate whether summer is associated with better IVF/ICSI outcomes in a large, national registry-based cohort study. Moreover, we aimed to elucidate whether seasonality and, indirectly, the highly varying sunlight length and the longer sun exposure during summer in the Nordic countries, is associated with IVF/ICSI outcome in terms of follicular development and embryo/endometrial interaction by separately analysing the outcomes per oocyte retrieval and per ET.

## Materials and methods

**Figure 1.**
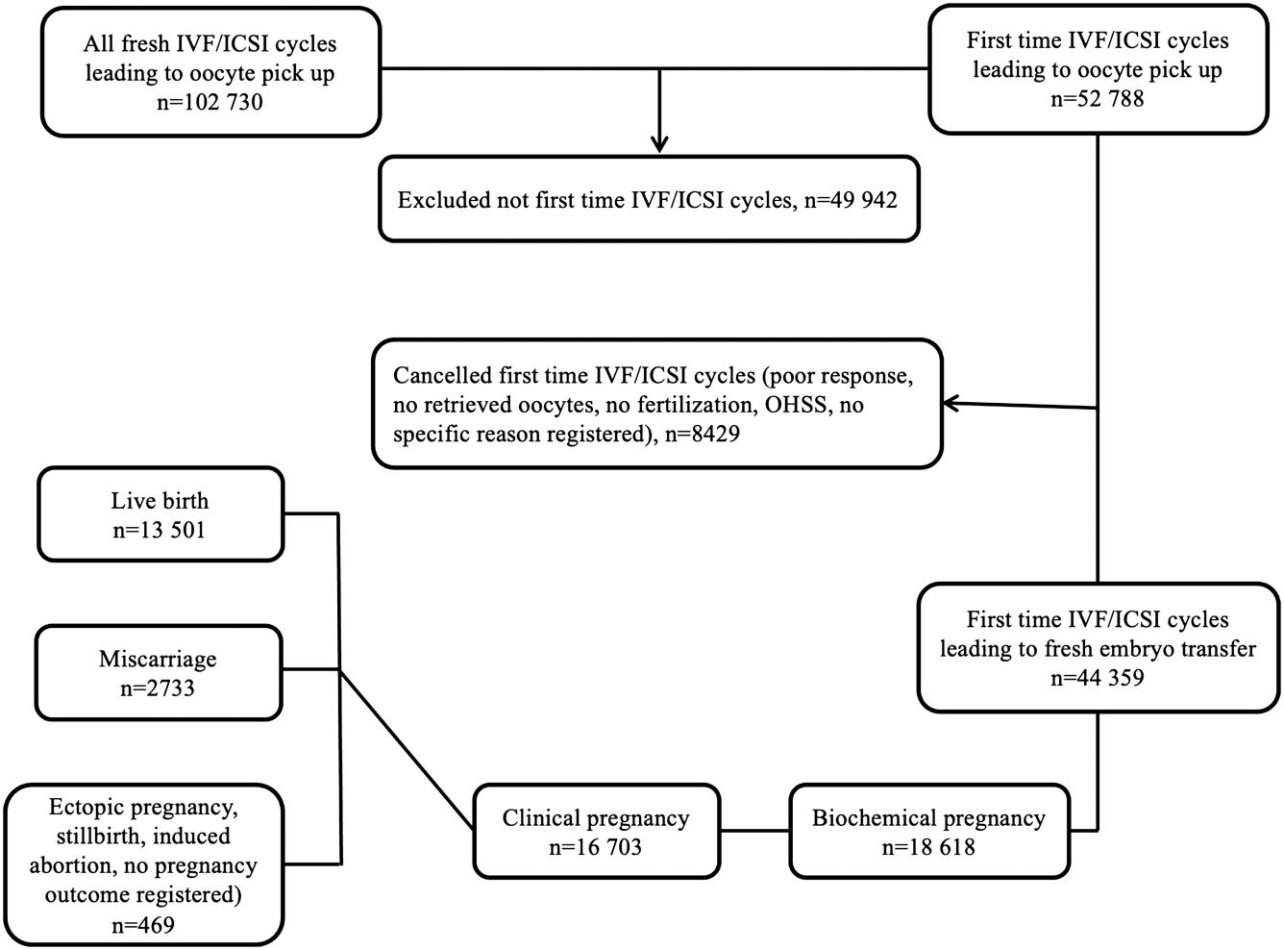
**Flowchart of the study design and patients included in a comparison of live birth rates in fresh IVF/ICSI cycles across the seasons.** The records are taken from the National Quality Registry of Assisted Reproduction (Q-IVF) in Sweden. OHSS, ovarian hyperstimulation syndrome.

Data were collected from the National Quality Registry of Assisted Reproduction (Q-IVF). All first-time fresh cycles resulting in oocyte retrieval and ET performed in Sweden from 2009 to 2018 were included. Cycles performed with donated and frozen oocytes, frozen/thawed embryos and cycles for fertility preservation were excluded. A flow chart of the study design and included patients is presented in Fig. 1. All Swedish IVF clinics, public as well as private, have reported their results to Q-IVF since 2007 and the results are publicly posted on the registry website. All patients are informed about the Q-IVF and may choose not to have their data included, although this is very rare.

Data collected from the Q-IVF included BMI of the woman, date of IVF treatment start, total gonadotrophin dose, type of GnRH protocol (agonist, antagonist), type of IVF treatment (fresh conventional IVF, fresh ICSI and fresh combination IVF/ICSI), date of oocyte retrieval, number of retrieved oocytes, date of ET, number of culture days of the transferred embryo(s), number of embryos transferred, urine-hCG test result, number of gestational sacs seen in transvaginal ultrasound in early pregnancy, miscarriages, live birth and date of delivery. A live birth was defined as the delivery of at least one live born child regardless of whether the pregnancy was singleton or multiple.

Women were treated with either a GnRH agonist or antagonist protocol and ovarian stimulation was performed with recombinant FSH or urinary-derived hMG. Standard techniques were used for oocyte retrieval and fertilization with conventional IVF or ICSI. In the vast majority of cycles, single embryo transfer (SET) was performed and no more than two embryos were ever transferred. ETs were performed with cleavage stage embryos (Days 2–3) or at the blastocyst stage.

The primary outcome was live birth rate, which was defined as the number of live births per oocyte retrieval and ET.

Biochemical pregnancy was defined as positive urine-hCG test. The biochemical pregnancy rate was defined as the number of women with positive urine-hCG test per ET. Clinical pregnancy was defined as the presence of at least one intrauterine gestational sac demonstrated by transvaginal ultrasound 7–9 weeks after ET. The clinical pregnancy rate was defined as the number of clinical pregnancies per ET. Miscarriage was defined as loss of pregnancy up to gestational week 22 and miscarriage rate was defined as the number of miscarriages per clinical pregnancy.

In order to differentiate the association between seasons and biochemical and clinical pregnancy rate, miscarriage rate and live birth rate, the cohort of women was subdivided into seasons based on the date that oocyte retrieval was performed. Seasons were defined before analysis of data according to the calendar definition of seasons for Sweden, each season lasting 3 months; spring, 1 March to 31 May; summer, 1 June to 31 August; autumn, 1 September to 30 November; and winter, 1 December to 28 or 29 February.

### Statistical analysis

Descriptive statistics are given as number and percentage for categorical variables and as mean with the SD and range for continuous variables. Logistic regression was used with summer as reference. Unadjusted and adjusted odds ratio (OR) with 95% CI are reported. Adjustment was performed for the following confounders: woman’s age, year of treatment, BMI, total FSH/hMG dose, type of treatment, fertilization type, embryonic stage at ET and number of embryos transferred. Owing to the amount of missing data on several of the confounders, multiple imputations were performed for the multivariable analysis. Missing data were assumed to be missing at random and 50 imputed datasets were generated with the Markov Chain Monte Carlo method using the expectation–maximization algorithm. Outcomes were not imputed and thus the procedure above was repeated separately for all first cycle oocyte retrievals and for each of the three outcome analyses (live birth rate, clinical pregnancy rate and miscarriage rate) for first cycles where ET was performed. Rubin’s rules were used to pool the results from the imputed datasets. *P*-values below 0.05 were considered statistically significant. All analyses were performed using SAS 9.4 for Windows (SAS Institute Inc., Cary, NC, USA).

### Ethical approval

The study was approved by the Swedish Ethical Review Authority, Dnr 2019-06551.

## Results

During the study period 2009–2018, a total of 102 730 fresh IVF/ICSI treatments leading to oocyte retrieval were started and, of these, 52 788 were first-time fresh IVF/ICSI cycles (51%). Among these IVF/ICSI cycles, there were 8429 cancelled treatments before fertilization (e.g. poor response after ovarian stimulation, no retrieved oocytes after oocyte retrieval) or before ET (e.g. threatening ovarian hyperstimulation syndrome resulting in freezing of all embryos, lack of embryos for transfer because of no fertilization or failed cleavage) (Fig. 1). Demographic and cycle characteristics for first fresh IVF/ICSI cycles are summarized in Tables I and II. ET was performed in 44 359 cycles, which represents 84% of all first-time cycles where oocyte retrieval have been performed (Fig. 1). In ∼80% of those cycles (range 80–81%), embryos were transferred at the cleavage stage, which was consistent among seasons. The majority of fresh ETs were performed with SET (89%), also consistent among seasons (range 88–91%) (Table II). Table III shows pregnancy outcomes for the first-time IVF/ICSI cycles.

**Table I:** Characteristics of all started first-time fresh IVF/ICSI cycles resulting in oocyte retrieval (national data Sweden 2009–2018).

	Spring	Summer	Autumn	Winter	Total
**Oocyte retrieval**	16 613 (31%)	7185 (14%)	16 737 (32%)	12 253 (23%)	52 788
**Age (years)**	33.3 ± 4.7 (18–48)	33.4 ± 4.7 (18–48)	33.3 ± 4.6 (19–47)	33.3 ± 4.7 (18–46)	
**BMI (kg/m^2^)**	24.2 ± 4.0 (13.2–49.0)	24.2 ± 4.0 (15.5–49.1)	24.2 ± 4.0 (14.7–42.0)	24.1 ± 4.0 (13.4–48.2)	
Missing data—BMI, n = 6589 women (12%)					
**Total FSH/hMG dose (IU)**	2021 ± 1121 (100–14 025)	2029 ± 1133 (100–8400)	1966 ± 1067 (100–10 312)	2012 ± 1115 (100–14 466)	
Missing data—total FSH/hMG dose, n = 6135 women (12%)					
**Type of COS protocol used**					
Agonist	5108 (31%)	2010 (28%)	4822 (29%)	3674 (30%)	15 614 (30%)
Antagonist	5543 (34%)	2449 (34%)	6127 (37%)	4079 (34%)	18 198 (35%)
Unspecified if agonist or antagonist	5863 (35%)	2677 (38%)	5697 (34%)	4424 (36%)	18 661 (35%)
Missing data—type of protocol used, n = 315 women (<1%)					
**Nr. of retrieved oocytes at oocyte retrieval**	9.7 ± 5.8 (0–54)	9.8 ± 5.9 (0–53)	9.8 ± 5.9 (0–57)	9.7 ± 5.8 (0–57)	
Missing data—number of retrieved oocytes, n = 27 women (<1%)					

COS, controlled ovarian stimulation. Categorical variables are presented as number (n) and percentage (%). Continuous variables are presented as mean ± SD (range). Missing data are shown in a separate row where applicable.

**Table II:** Characteristics of all started first-time fresh IVF/ICSI cycles resulting in embryo transfer (national data Sweden 2009–2018).

	Spring	Summer	Autumn	Winter	Total
**ET**	14 015 (32%)	6018 (14%)	14 068 (32%)	10 258 (23%)	44 359
**Age (years)**	33.4 ± 4.7 (18–48)	33.5 ± 4.7 (20–48)	33.4 ± 4.6 (19–46)	33.4 ± 4.6 (18–46)	
**BMI (kg/m^2^)**	24.1 ± 3.9 (13.2–49.0)	24.2 ± 4.0 (15.8–49.1)	24.2 ± 4.0 (14.7–42.0)	24.1 ± 3.9 (13.4–48.2)	
Missing data—BMI, n = 5497 women (12%)					
**Total FSH/hMG dose (IU)**	2014 ± 1096 (100–14 025)	2026 ± 1123 (100–8250)	1958 ± 1043 (100–9750)	1993 ± 1082 (100–14 466)	
Missing data—total FSH/hMG dose, n = 5318 women (12%)					
**Type of COS protocol used**					
Agonist	4492 (32%)	1765 (30%)	4240 (30%)	3209 (32%)	
Antagonist	4485 (32%)	1973 (33%)	4964 (36%)	3297 (32%)	
Unspecified if agonist or antagonist	4951 (36%)	2238 (37%)	4783 (34%)	3689 (36%)	
Missing data—type of COS protocol used, n = 273 women (<1%)					
**Number of retrieved oocytes at** oocyte retrieval	9.4 ± 5.0 (1–44)	9.4 ± 5.0 (1–34)	9.5 ± 5.1 (1–40)	9.4 ± 5.0 (1–38)	
Missing data—number of retrieved oocytes, n = 24 women (<1%)					
**Type of fertilization**					
Conventional IVF	8316 (62%)	3478 (61%)	8304 (62%)	6029 (61%)	26 127 (59%)
ICSI	5170 (38%)	2245 (39%)	5188 (38%)	3847 (39%)	16 450 (37%)
Combination IVF/ICSI	21 (<1%)	9 (<1%)	18 (<1%)	24 (<1%)	72 (<1%)
Missing data—type of fertilization, n = 1710 women (4%)					
**Embryonic stage at ET (days)**					
Cleavage	11 264 (80%)	4843 (81%)	11 222 (80%)	8163 (80%)	35 492 (80%)
Blastocyst	2734 (20%)	1169 (19%)	2828 (20%)	2080 (20%)	8811 (20%)
Missing data—Embryonic stage at ET, n = 56 women (<1%)					
**Number of embryos transferred**					
SET	12 342 (88%)	5368 (89%)	12 756 (91%)	9072 (88%)	39 538 (89%)
DET	1658 (12%)	644 (11%)	1297 (9%)	1177 (12%)	4776 (11%)
Missing data—number of embryos transferred, n = 45 women (<1%)					

ET, embryo transfer; COS, controlled ovarian stimulation; SET, single embryo transfer; DET, double embryo transfer. Categorical variables are presented as number (n) and percentage (%). Continuous variables are presented as mean ± SD (range). Missing data are shown in a separate row when applicable.

**Table III:** Outcome for all started fresh first-time IVF/ICSI cycles resulting in oocyte retrieval and embryo transfer (national data Sweden 2009–2018).

	Spring	Summer	Autumn	Winter	Total
**Oocyte retrieval, n**	16 613	7185	16 737	12 253	52 788
**ET, n**	14 015	6018	14 068	10 258	44 359
**Live birth, n (per oocyte retrieval, %)**	4315 (26%)	1741 (24%)	4295 (26%)	3150 (26%)	13 501 (26%)
**Live birth, n (per ET, %)**	4315 (31%)	1741 (29%)	4295 (31%)	3150 (31%)	13 501 (30%)
**Biochemical pregnancy, n (per ET, %)**	5906 (42%)	2435 (40%)	5947 (42%)	4330 (42%)	18 618 (42%)
**Clinical pregnancy, n (per ET, %)**	5316 (38%)	2183 (36%)	5316 (38%)	3888 (38%)	16 703 (38%)
**Miscarriage, n (per clinical pregnancy, %)**	862 (16%)	386 (18%)	860 (16%)	625 (16%)	2733 (16%)

ET, embryo transfer; Live birth, delivery of at least one live born child regardless of whether the pregnancy was singleton or multiple; Biochemical pregnancy, positive urine-hCG test; Clinical pregnancy, presence of at least one intrauterine gestational sac demonstrated by transvaginal ultrasound 7–9 weeks after embryo transfer; Miscarriage, loss of pregnancy up to gestational week 22.

Live birth rates per oocyte retrieval (n = 13 501) ranged between 24% and 26% among the seasons. Autumn, winter and spring had significantly higher live birth rates per oocyte retrieval compared with summer in the unadjusted analyses (autumn, OR 1.08, 95% CI 1.02–1.16, *P *=* *0.02; winter, OR 1.08, 95% CI 1.01–1.16, *P *=* *0.02; and spring, OR 1.10, 95% CI 1.03–1.17, *P *=* *0.005). After adjustment for confounders, spring had a significantly higher live birth rate per oocyte retrieval compared with summer (adjusted OR 1.08, 95% CI 1.02–1.16, *P *=* *0.02). No significant association was seen when autumn and winter were compared with summer (autumn, adjusted OR 1.06, 95% CI 1.0–1.14, *P *=* *0.06 and winter, adjusted OR 1.07, 95% CI 1.0–1.15, *P *=* *0.05), respectively (Table IV).

**Table IV:** Live birth rate per oocyte retrieval, first-time fresh IVF/ICSI cycles (national data Sweden 2009–2018).

Live birth rate per oocyte pick-up				
	Crude OR (95% CI)	*P*-value	Adjusted OR (95% CI)	*P*-value
Summer (reference)	1		1	
Autumn	1.08 (1.02, 1.16)	0.02	1.06 (1.0, 1.14)	0.06
Winter	1.08 (1.01, 1.16)	0.02	1.07 (1.0, 1.15)	0.05
Spring	1.10 (1.03, 1.17)	0.005	1.08 (1.02, 1.16)	0.02

Crude and adjusted odds ratio (OR) for live birth rate with 95% CI for all performed first cycles. Adjusted for woman’s age, year of treatment, BMI, total FSH/hMG dose and type of treatment. Analysis was performed by logistic regression with use of multiple imputations. The seasonal influence was calculated by comparing summer with the other seasons.

Live birth rates per ET (n = 13 501) ranged between 29% and 31% among the seasons. Autumn, winter and spring had significantly higher live birth rates per ET compared with summer in the unadjusted analyses (autumn, OR 1.08, 95% CI 1.02–1.16, *P *=* *0.02; winter, OR 1.09, 95% CI 1.02–1.17, *P *=* *0.02; and spring, OR 1.09, 95% CI 1.02–1.17, *P *=* *0.008). After adjustment for confounders, autumn and spring had significantly higher live birth rates per ET compared with summer (autumn, adjusted OR 1.09, 95% CI 1.01–1.16, *P *=* *0.02 and spring, adjusted OR 1.08, 95% CI 1.01–1.16, *P *=* *0.04), respectively. No significant association was seen when winter was compared with summer (adjusted OR 1.07, 95% CI 1.0–1.15, *P *=* *0.05) (Table V).

**Table V:** Clinical pregnancy and live birth rates per embryo transfer and miscarriage rate per clinical pregnancy, first-time fresh IVF/ICSI cycles (national data Sweden 2009–2018).

	Crude OR (95% CI)	*P*-value	Adjusted OR (95% CI)	*P*-value
**Clinical pregnancy rate per embryo transfer**				
Summer (reference)	1		1	
Autumn	1.07 (1.0, 1.14)	0.05	1.05 (0.99, 1.12)	0.12
Winter	1.07 (1.0, 1.14)	0.04	1.06 (0.99, 1.13)	0.09
Spring	1.07 (1.01, 1.14)	0.03	1.06 (1.0, 1.13)	0.06
**Miscarriage rate per clinical pregnancy**				
Summer (reference)	1		1	
Autumn	0.91 (0.79, 1.03)	0.14	0.91 (0.79, 1.04)	0.17
Winter	0.90 (0.78, 1.03)	0.12	0.93 (0.81, 1.06)	0.29
Spring	0.90 (0.79, 1.03)	0.13	0.91 (0.79, 1.05)	0.21
**Live birth rate per embryo transfer**				
Summer (reference)	1		1	
Autumn	1.08 (1.02, 1.16)	0.02	1.09 (1.01, 1.16)	0.02
Winter	1.09 (1.02, 1.17)	0.02	1.07 (1.0, 1.15)	0.05
Spring	1.09 (1.02, 1.17)	0.008	1.08 (1.01, 1.16)	0.04

Crude and adjusted odds ratio (OR) for clinical pregnancy-, miscarriage- and live birth rates with 95% CI for all performed first IVF/ICSI cycles. Adjusted for woman’s age, year of treatment, BMI, total FSH/hMG dose, type of treatment, fertilization type, embryo age and number of embryos transferred. Analysis was performed by logistic regression with multiple imputations. The seasonal influence was calculated by comparing summer with the other seasons.

Clinical pregnancy rates varied between 36% and 38% among seasons and no significant association was seen between summer and the other seasons in the adjusted analyses. Miscarriage rate varied between 16% and 18% among seasons and no association was seen between summer and the other seasons in the adjusted analyses (Table V).

## Discussion

This large registry-based cohort study included 52 788 first-time fresh IVF/ICSI cycles where we examined the association between seasons and the rates of live birth, clinical pregnancy and miscarriage after IVF/ICSI. Our main findings were a slightly but significantly lower live birth rate per oocyte retrieval when IVF/ICSI treatment was performed in summer compared to spring and also per ET when treatment was performed in summer compared to autumn and spring. Live birth rates per oocyte retrieval ranged between 24% and 26% and live birth rates per ET between 29% and 31%. The miscarriage rate, ranging between 16% and 18%, is in line with large registry data from the USA and Europe (Centers for Disease Control and Prevention, 2021; Wyns *et al.*, 2021). There was no significant association between season and clinical pregnancy rate and miscarriage rate.

Several small studies have shown better pregnancy outcomes when IVF/ICSI treatment was performed during the period of the year with increased daylight length (Rojansky *et al.*, 2000; Wood *et al.*, 2006; Braga *et al.*, 2012; Vandekerckhove *et al.*, 2016; Farland *et al.*, 2020) and one study has shown better pregnancy outcome during the summer months when sunlight peaks (Wood *et al.*, 2006). It has been hypothesized that serum vitamin D, levels of which are associated with sun exposure, may have a role in human natural conception rates. Some studies have supported a role of vitamin D in reproductive physiology (Malloy *et al.*, 2009; Merhi *et al.*, 2012) and an association between vitamin D sufficiency and higher success rates after IVF has been reported (Iliuta et al., 2022). However, a recent systematic review and meta-analysis showed no association between serum vitamin D levels and IVF/ICSI outcomes (Cozzolino *et al.*, 2020).

The results of this study do not support the hypothesis that longer sunlight exposure during summer, with expected higher serum vitamin D levels, is associated with better pregnancy outcomes after IVF/ICSI. This is in line with other smaller studies that showed no seasonal association with pregnancy outcomes after ART (Fleming *et al.*, 1994; Gindes *et al.*, 2003; Revelli *et al.*, 2005; Wunder *et al.*, 2005; Kirshenbaum *et al.*, 2018; Liu *et al.*, 2019). In fact, our results showed slightly lower live birth rates when treatment was performed in summer compared with autumn and spring. However, although this finding was statistically significant, the magnitude of these differences was small and probably of no clinical impact.

The number of IVF/ICSI treatments performed during summer was lower compared to the other seasons owing to the summer vacation, with closed IVF clinics, in Sweden and thus limited resources during the summer months.

Major strengths of the present study are the large sample size and inclusion of a complete national cohort during a given time period, without any selection. Furthermore, we included only the first cycle per patient, thus eliminating dependence between cycles from the same patient. The number of included fresh IVF cycles in previous studies ranged between 577 and 13 223 (Rojansky *et al.*, 2000; Gindes *et al.*, 2003; Revelli *et al.*, 2005; Wunder *et al.*, 2005; Wood *et al.*, 2006; Braga *et al.*, 2012; Vandekerckhove *et al.*, 2016; Kirshenbaum *et al.*, 2018; Liu *et al.*, 2019) and only three studies reported results from the first fresh IVF cycle, with low sample sizes (Rojansky *et al.*, 2000; Revelli *et al.*, 2005; Wood *et al.*, 2006). Another strength of the study is that we have chosen to analyse the association of seasons with the outcome of fresh IVF/ICSI cycles leading to oocyte retrieval to assess the combined effect on fertilization, embryo development and endometrial receptivity (without isolating the effect on endometrial receptivity when only including cycles where ET was performed). Another strength is that we were able to adjust for several relevant confounders.

Possible limitations are the retrospective design of the study and residual confounding. Another limitation is the inclusion of only first cycles, thus making the study population smaller. The use of a generalized estimating equation model would have made it possible to include all started fresh IVF/ICSI cycles since it allows for correction for any dependence between cycles within women.

In conclusion, by analysing a large and complete cohort of fresh IVF/ICSI cycles, we showed that performing treatment in summer is not associated with a better pregnancy outcome. In contrast, other seasons showed a slightly higher live birth rate compared with summer; however, this difference is unlikely to have clinical value. We suggest that season should not be taken into consideration when planning and performing IVF/ICSI.

## Data Availability

Data are available on request. The data underlying this article will be shared on reasonable request to the first or corresponding authors.
